# A Transcriptome Approach Toward Understanding Fruit Softening in Persimmon

**DOI:** 10.3389/fpls.2017.01556

**Published:** 2017-09-12

**Authors:** Jihye Jung, Sang Chul Choi, Sunghee Jung, Byung-Kwan Cho, Gwang-Hwan Ahn, Stephen B. Ryu

**Affiliations:** ^1^Department of Biological Sciences, Korea Advanced Institute of Science and Technology Daejeon, South Korea; ^2^Plant Systems Engineering Research Center, Korea Research Institute of Bioscience and Biotechnology Daejeon, South Korea; ^3^Department of Biosystems and Bioengineering, KRIBB School of Biotechnology, Korea University of Science and Technology Daejeon, South Korea; ^4^Sweet Persimmon Research Institute, Gyeongsangnam-do Agricultural Research and Extension Services Gimhae, South Korea

**Keywords:** *Diospyros kaki*, persimmon softening, *de novo* RNA sequencing, ethylene, transcriptional events

## Abstract

Persimmon (*Diospyros kaki* Thunb.), which is a climacteric fruit, softens in 3–5 weeks after harvest. However, little is known regarding the transcriptional changes that underlie persimmon ripening. In this study, high-throughput *de novo* RNA sequencing was performed to examine differential expression between freshly harvested (FH) and softened (ST) persimmon fruit peels. Using the Illumina HiSeq platform, we obtained 259,483,704 high quality reads and 94,856 transcripts. After the removal of redundant sequences, a total of 31,258 unigenes were predicted, 1,790 of which were differentially expressed between FH and ST persimmon (1,284 up-regulated and 506 down-regulated in ST compared with FH). The differentially expressed genes (DEGs) were further subjected to KEGG pathway analysis. Several pathways were found to be up-regulated in ST persimmon, including “amino sugar and nucleotide sugar metabolism.” Pathways down-regulated in ST persimmon included “photosynthesis” and “carbon fixation in photosynthetic organisms.” Expression patterns of genes in these pathways were further confirmed using quantitative real-time RT-PCR. Ethylene gas production during persimmon softening was monitored with gas chromatography and found to be correlated with the fruit softening. Transcription involved in ethylene biosynthesis, perception and signaling was up-regulated. On the whole, this study investigated the key genes involved in metabolic pathways of persimmon fruit softening, especially implicated in increased sugar metabolism, decreased photosynthetic capability, and increased ethylene production and other ethylene-related functions. This transcriptome analysis provides baseline information on the identity and modulation of genes involved in softening of persimmon fruits and can underpin the future development of technologies to delay softening in persimmon.

## Introduction

Seven of the approximately 190 known species of persimmon (*Diospyros*) are grown commercially for fruit production, the most widely cultivated of which is *Diospyros kaki*. The *D. kaki* “Fuyu” sweet persimmon is a pollination-constant and non-astringent cultivar (Ito, 1971). After harvest, persimmon fruits often undergo rapid softening, darkening, and browning, resulting in short shelf life (Hobson, 1981). Persimmons are cultivated in sub-tropical and warm temperate regions, and their production is limited to certain areas in countries such as China, Korea, Japan, and Brazil (Mowat et al., 1995; FAOSTAT, 2013). The short shelf life is a barrier to successful exportation of persimmon fruit to other countries, and extending shelf life is economically desirable.

Persimmon produces ethylene during fruit ripening and is classified as a climacteric plant based on its pattern of respiration and ethylene production (Biale, 1981). Ethylene causes fruit softening by inducing the expression of cell wall degradation genes, resulting in a modified fruit texture (Mattoo and Suttle, 1991; Nakano et al., 2003; Nishiyama et al., 2007). Aminocyclopropane-1-carboxylic acid (ACC) synthase and ACC oxidase (ACO) are known key enzymes for synthesizing the ethylene and silencing of these genes inhibited ripening in tomato (Oeller and Min-Wong, 1991; Fluhr et al., 1996). Chemical treatment such as 1-methylcyclopropene (1-MCP), an inhibitor of ethylene perception, also delays fruit ripening (Mattoo and Suttle, 1991; Vilas-Boas and Kader, 2007). Therefore, ethylene perception in addition to ethylene production is a major cause of fruit softening.

Little is known regarding the genes associated with fruit softening in persimmon. Ethylene biosynthesizing genes, ACC synthase (*DkACS1*, -*2*, and -*3*) and ACC oxidase (*DkACO1* and -*2*), were known in persimmon and the expression of these genes is temporally and spatially regulated during fruit softening (Nakano et al., 2003; Ortiz et al., 2006). Two persimmon homologs of *Arabidopsis* ethylene receptor genes, namely, *DkERS1* and *DkETR2*, exhibited induced expression during softening (Pang et al., 2007). Expressed sequence tag (EST) analysis showed that cell wall degradation-related genes such as those encoding basic chitinase and endochitinase were also induced during later stages of persimmon fruit development (Nakagawa et al., 2008).

Transcriptional analysis of fruit ripening was previously examined in several species, including bayberry, tomato, and watermelon, and specific transcripts were linked to increases in fruit quality and reductions in softening (Guo et al., 2011; Feng et al., 2012; Lee et al., 2012). Softening-related expression profiles differed between fruit species (Brummell and Harpster, 2001). Previous transcriptional analysis of persimmon examined expression changes after ethanol treatment or under anoxic conditions (Luo et al., 2014; Min et al., 2014). However, to date, fruit softening in persimmon has only been examined at the individual gene level rather than with comprehensive transcriptional analysis (Hou et al., 2015).

*De novo* transcriptome assembly is a powerful technique for assessing transcription in the absence of a reference genome (Haas et al., 2013). In this study, *de novo* RNA sequencing (RNA-seq) using Illumina HiSeq 2000 technology was used to generate transcriptomes from freshly harvested (FH) and softened (ST) persimmon fruit peels. This investigation identified several genes involved in persimmon fruit softening and provides insights into the molecular events underlying the regulation of persimmon fruit softening.

## Materials and methods

### Plant material and sample preparation

Persimmon fruits were harvested and stored in constant conditions (22°C, 60% humidity). Persimmon fruit peels (*n* = 5) collected at 5 days after harvest (DAH) were used as the FH sample for RNA-seq. Peels were harvested for the ST sample when 80% of the stored fruits had softened. One and two (a total of three) FH and ST samples were collected at two different years, respectively. Persimmon peel samples were also taken at 1, 13, 25, and 34 DAH for gene expression analysis with quantitative real-time RT-PCR (qRT-PCR) to verify RNA-seq analysis results. All samples were frozen in liquid nitrogen immediately upon harvest.

### RNA isolation, cDNA library construction, and RNA-seq

Total RNA was extracted from persimmon fruit peels using cetyl trimethyl ammonium bromide (CTAB) buffer [2.0% CTAB, 1.4 M NaCl, 20 mM EDTA, pH 8.0, 100 mM Tris-HCl, pH 8.0, 2.0% polyvinylpyrrolidone (PVP40), 1% β-mercaptoethanol] after sample grinding in liquid nitrogen. Total RNA was purified using an RNeasy mini prep kit (Qiagen) and treated with DNase I (Qiagen) to remove residual DNA. The cDNA libraries for RNA-seq were prepared using an Illumina TruSeq DNA/RNA library preparation kit according to the manufacturer's protocol. Briefly, RNA was fragmented to an average length of 200–300 bp by Mg^2+^-catalyzed hydrolysis and then reverse transcribed to cDNA with random priming.

The cDNA was then subjected to end repair, adaptor ligation, size selection, and PCR amplification. Each cDNA library was 101 bp paired-end sequenced using an Illumina HiSeq 2000 sequencer according to the manufacturer's protocols. The whole transcriptome sequence raw data were deposited in the NCBI database (https://www.ncbi.nlm.nih.gov/sra) with accession numbers SRR4345331 and SRR4345332 for FH and ST, respectively. The assembled sequence database was also deposited in the NCBI database with accession number GFUF01000000.

### Sequence preprocessing and *de novo* assembly

Raw 101 bp paired-end reads were filtered by quality score (Q ≥ 20) using SolexaQA (Cox et al., 2010). Trimming resulted in reads with a mean length of 85.61 bp across all samples and a minimum length of 25 bp. *De novo* assembly of trimmed reads was carried out using the de Bruijn graph-based assemblers Velvet (v1.2.07) (Zerbino and Birney, 2008) and Oases (v0.2.08) (Schulz et al., 2012). To optimize data assembly, *de novo* assembly was performed using various hash lengths (*k*-mers) in the 51–75 range.

### Validation of unigene assembly and annotation

Unigene transcripts for persimmon were assembled from merged total reads from both the FH and ST mRNA samples. Translated transcripts were validated by comparison with sequences in the Phytozome database (http://www.phytozome.net) using BLASTx (*E* ≤ 1e^−10^) (Goodstein et al., 2012). Protein sequences with the highest sequence similarities were retrieved for further study.

### Functional annotation

Unigenes were functionally annotated using The Arabidopsis Information Resource (TAIR) database and subjected to KEGG (http://www.genome.jp/kegg/) pathway analysis (Kanehisa et al., 2004). In addition, translated transcripts were compared with the KOG (http://www.ncbi.nlm.nih.gov/COG/) protein database using BLASTx (*E* ≤ 1e^−10^) (Tatusov et al., 2003), and the best-matching results were selected to annotate the unigenes.

### Short read mapping and analysis of differentially expressed genes (DEGs)

Reads for each sequence were mapped to the assembled unigenes using bowtie2 software with default parameters (Langmead and Salzberg, 2012). After counting the number of reads mapped to each transcript, expression values were normalized using DESeq method (Anders and Huber, 2010). DEGs that were differentially expressed between the FH and ST samples were determined from three replicates samples using the binomial test method with expression levels log_2_ > 1 or < −1, and false discovery rate (FDR) < 0.01. KEGG pathway analysis of DEGs was conducted with TAIR ID using DAVID (Huang et al., 2008) with a two-count option and *p* < 0.35.

### Expression analysis using qRT-PCR

First strand cDNA for qRT-PCR was synthesized from total RNA (0.3 μg) using SuperScript III transcriptase (Invitrogen) according to the manufacturer's instructions. Expression analysis was performed using a CFX96 Real-time PCR detection system (Bio-Rad) with SYBR green fluorescent dye (Enzynomics). The thermal cycling program was 95°C for 5 min, 40 cycles of 95°C for 15 s, 55, 57, or 59°C for 30 s, and 72°C for 30 s. The *D. kaki Actin* gene (accession no. AB473616) served as a reference for expression normalization. PCR specificity was confirmed by electrophoresis gel, amplicon size and DNA sequencing of PCR products (Bustin et al., 2009). The ΔΔCt method was used to calculate gene expression (Livak and Schmittgen, 2001). For convenient comparisons, the expression levels were presented as relative values compared to those of the day 1 samples (expression level = 1). Gene-specific primer sequences are listed in Table S7.

### Ethylene quantification analysis

Sequence of the genes were deposited in the NCBI database (https://www.ncbi.nlm.nih.gov/sra) with accession numbers in Table S8. The rate of ethylene production by whole persimmon fruits at 1, 13, 25, and 34 DAH was measured by enclosing two samples in 2 L airtight containers for 1 h at 22°C. Headspace gas (0.2 mL) was withdrawn and injected into a gas chromatograph (model GC-4CMPF, Shimadzu, Kyoto) equipped with a flame ionization detector and a 2,000 × 3 mm aluminum oxide column. Ethylene production was presented as nL·g FW^−1^·h^−1^, and five replicate measurements were performed.

## Results

### *De novo* RNA-seq analysis of FH or ST persimmon

Persimmon fruits soften after harvesting as a result of metabolic processes induced by transcriptional changes. RNA-seq was used to examine differential gene expression between peel samples from FH and ST persimmon. In total, 289,161,674 raw reads and 29.1 Gbp sequence were obtained from all samples using the Illumina HiSeq 2000 platform (Table S1). To assess sequence data quality, raw reads were trimmed and sorted by length using the Dynamic Trim and Length Sort programs in the SolexaQA package, which is used (Cox et al., 2010). Of the raw reads, 89.74% had high-quality score thresholds of Q ≥ 20 and read length >25 bp. In total, 259,483,704 high-quality reads with 23,283,962,364 bases were obtained.

Processed sequence reads were assembled *de novo* into transcripts using Velvet and Oases based on the de Bruijn graph algorithm (Zerbino and Birney, 2008; Schulz et al., 2012). To optimize data assembly, *de novo* assembly was performed using various hash lengths (*k*-mers) in the 51–75 range. The assembled transcripts were combined using Velvet and Oases with *k*-mer = 61, resulting in 94,856 extended transcripts with lengths ≥200 bp. After removal of highly similar redundant transcripts, 31,258 unigene transcripts were defined and used for further analyses (Table 1). The unigenes comprised 30,524,524 bp in total and had an average length of 976 bp, a maximum length of 10,510 bp, and N50 of 1,695 bp (Table 1). The majority of the unigenes (27,046; 86.53%) were 0.2–2 kb in length (Figure S1).

**Table 1:** Statistical summary of *de novo* assembly of *D. kaki transcripts*.

	**Total number of transcripts**	**Length (bp) of transcripts**				
		**Total length**	**Min**	**Max**	**Average**	**N50**
Total transcripts	94,856	145,748,163	200	10,510	1,536	2,190
Unigenes	31,258	30,524,524	200	10,510	976	1,695

### Functional annotation of assembled persimmon transcripts

The 31,258 assembled unigenes were searched against the GenBank non-redundant database using BLASTx (*E* ≤ 1e^−10^) (http://www.ncbi.nlm.nih.gov/). Annotated proteins were identified in the NR database with hits against 16,806 (53.76%) of the unigenes (Table 2). The reliability of the assembled transcripts was assessed by BLASTx comparison of the 31,258 unigenes against the plant-specific Phytozome database (http://www.phytozome.net). Phytozome provides a view of the evolutionary history of each plant gene at the level of sequence, gene structure, gene family, and genome organization, and provides sequence and functional annotation information for each gene (Goodstein et al., 2012). Of the 31,258 unigenes, 18,424 (58.94%) transcripts shared sequence similarity with genes from 41 plant species in Phytozome (*E* ≤ 1e^−10^; Table S2). The plant species with the highest numbers of BLASTx hits were *Vitis vinifera* (3,720 transcripts; 20.19%), *Theobroma cacao* (2,037 transcripts; 11.06%), *Manihot esculenta* (1,324 transcripts; 7.19%), *Prunus persica* (1,229 transcripts; 6.67%), *Populus trichocarpa* (1,122 transcripts; 6.09%), and *Solanum tuberosum* (1,053 transcripts; 5.72%) (Figure S2). These results showed that the assembled *D. kaki* transcripts shared similarity with transcripts from several reference species that were not evolutionarily close to *D. kaki*.

**Table 2:** Annotation summary of the assembled transcripts.

**Database**	**Number of assembled transcripts (hits percentage)**
Total unigenes	31,258 (100.00%)
Nr (viridiplantae)	16,806 (53.76%)
Phytozome	18,424 (58.94%)
KOG	16,574 (53.02%)
KEGG	2,115 (6.76%)
Total annotation	18,446 (59.01%)

**Figure 1:**
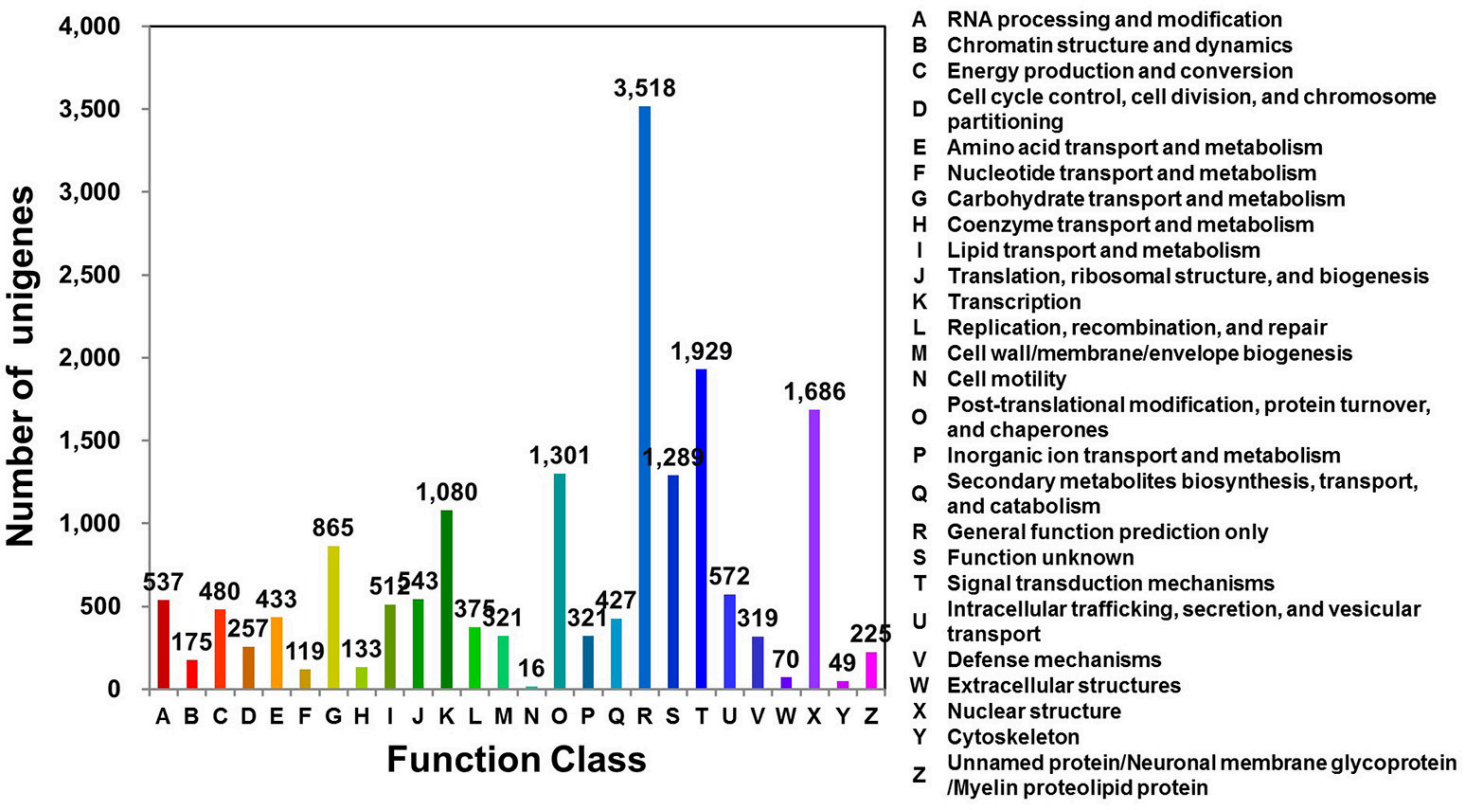
KOG functional classification of *D. kaki*. The unigenes of persimmon fruit peels were aligned to the KOG database to predict and classify possible functions. A total of 16,574 unigenes were assigned to functional orthologs.

Clusters of orthologous groups for eukaryotic genome (KOG; http://www.ncbi.nlm.nih.gov/COG/) analysis can be used for functional annotation of transcriptomes and newly sequenced genomes (Tatusov et al., 2003). *D. kaki* unigenes were matched to sequences in the KOG database using BLASTx (*E* ≤ 1e^−10^). Of the 31,258 unigenes, 16,574 (53.02%) were assigned to 26 KOG categories (Figure 1; Table S3). Of these 26 categories, the cluster for “general function prediction only” was the largest group (3,518; 21.23%), followed by “signal transduction mechanisms” (1,929; 11.64%), and “nuclear structure” (1,686; 10.17%).

**Figure 2:**
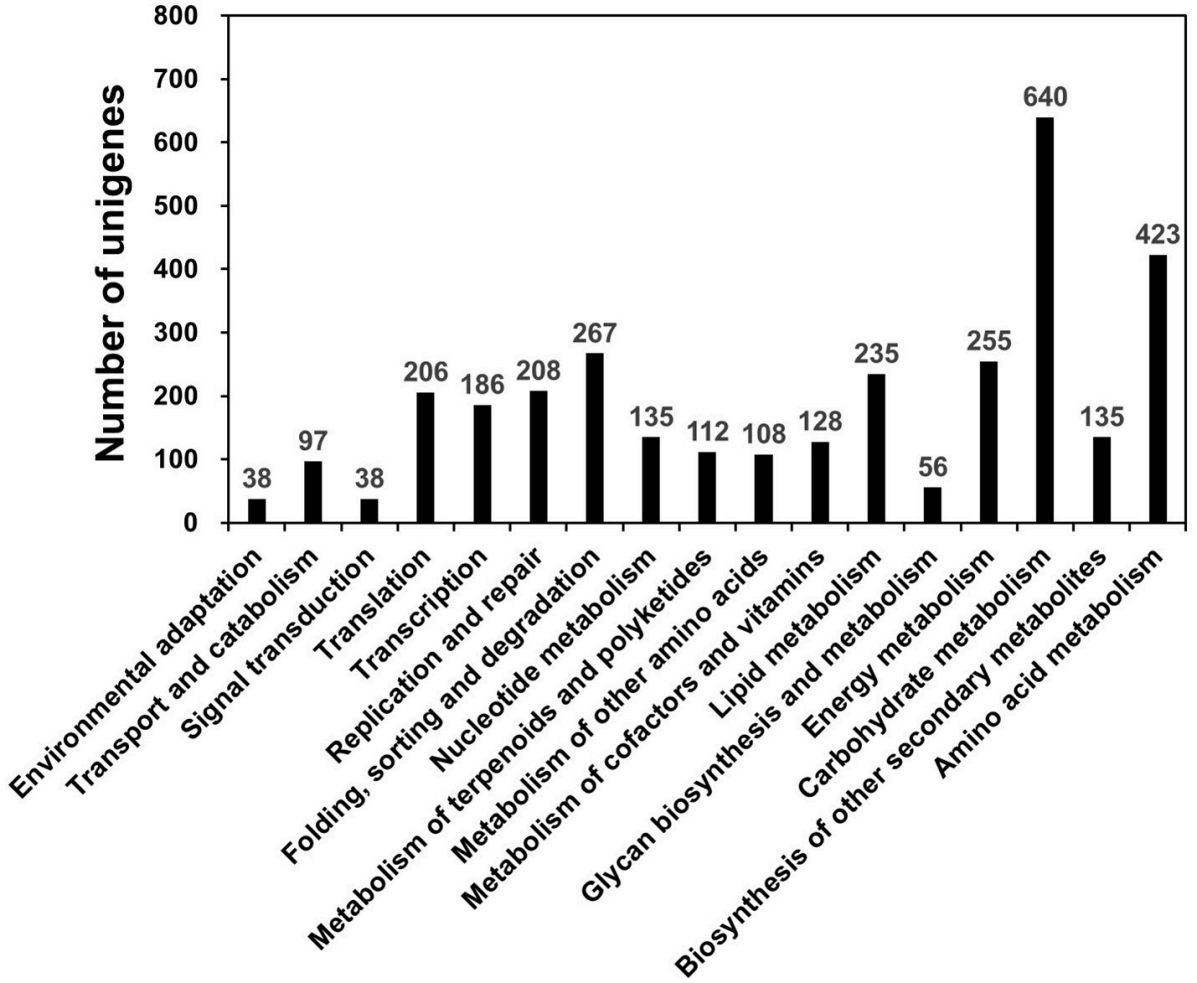
KEGG pathway mapping for *D. kaki*. The unigenes of persimmon fruit peels were functionally classified according to metabolic function.

KEGG, an alternative functional gene annotation system, examines molecular interaction networks. KEGG pathway analysis was performed with the 31,258 unigenes using the KEGG Automatic Annotation Server with TAIR as a reference (Kanehisa et al., 2004). KEGG mapped 2,115 unigenes (6.76%) to 17 predicted pathways (Figure 2). Of the five categories identified, the largest was “Metabolism” (2,115 unigenes), which contained “carbohydrate metabolism” (640 unigenes), “amino acid metabolism” (423 unigenes), “energy metabolism” (255 unigenes), “lipid metabolism” (235 unigenes), and other sub-categories (Figure 2; Table S4). The category “Genetic Information Pathways” also contained a large number of unigenes (867), namely, “translation” (206 unigenes), “transcription” (186 unigenes), “replication and repair” (208 unigenes), and “folding, sorting, and degradation” (267 unigenes) (Figure 2; Table S4). The major categories of “Cellular Processes,” “Environmental Information Processing,” and “Organismal Systems” contained 97, 38, and 38 unigenes, respectively (Figure 2; Table S4).

### Biochemical pathways of DEGs in FH and ST persimmon

**Figure 3:**
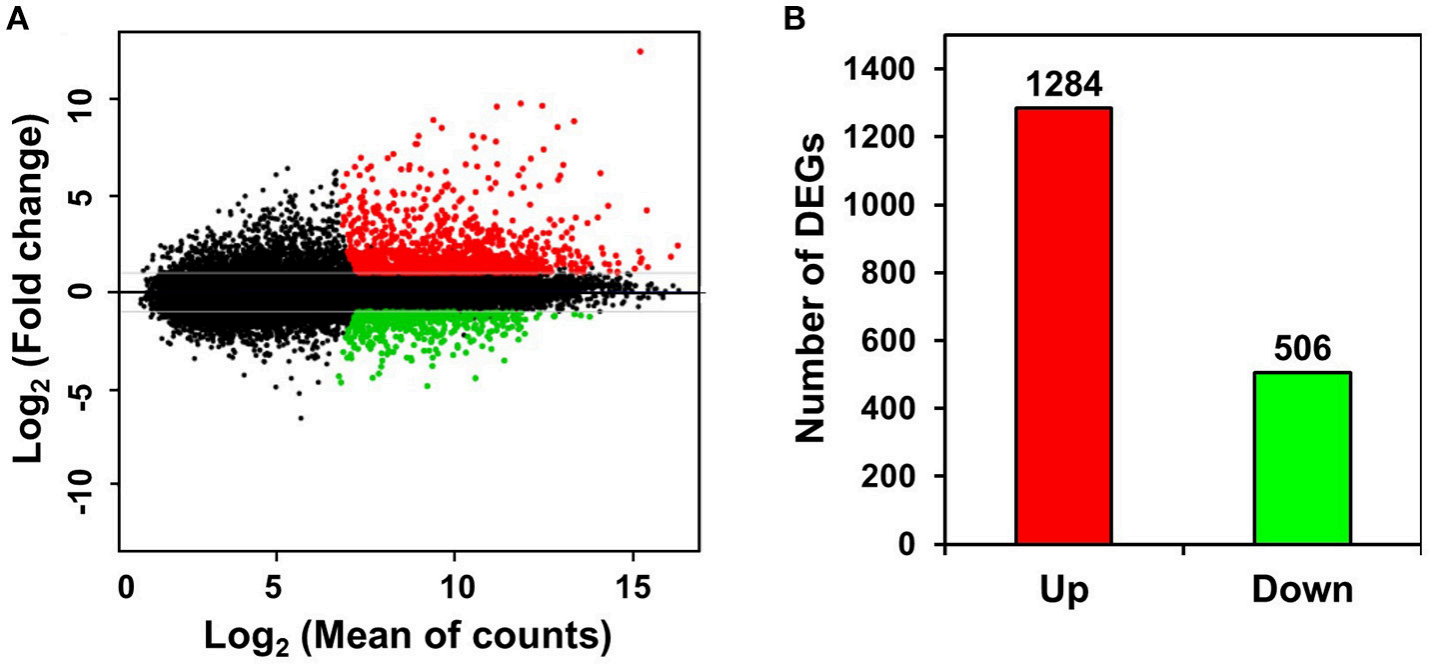
Differential gene expression between freshly harvested (FH) and softened (ST) persimmon. **(A)** MA plot of DEGs between FH and ST. Genes up- and down-regulated in ST are shown in red and green, respectively. **(B)** Numbers of up- and down-regulated genes in ST compared with FH persimmon.

Unigenes with >2-fold expression differences between ST and FH persimmon were defined as DEGs. DEGs were considered significant at FDR adjusted *p* < 0.01 (Figure 3A; Table S5). Of the 1,790 DEGs identified, 1,284 unigenes exhibited elevated expression and 506 unigenes exhibited lower expression in ST compared with FH persimmon (Figure 3B; Table S5).

**Figure 4:**
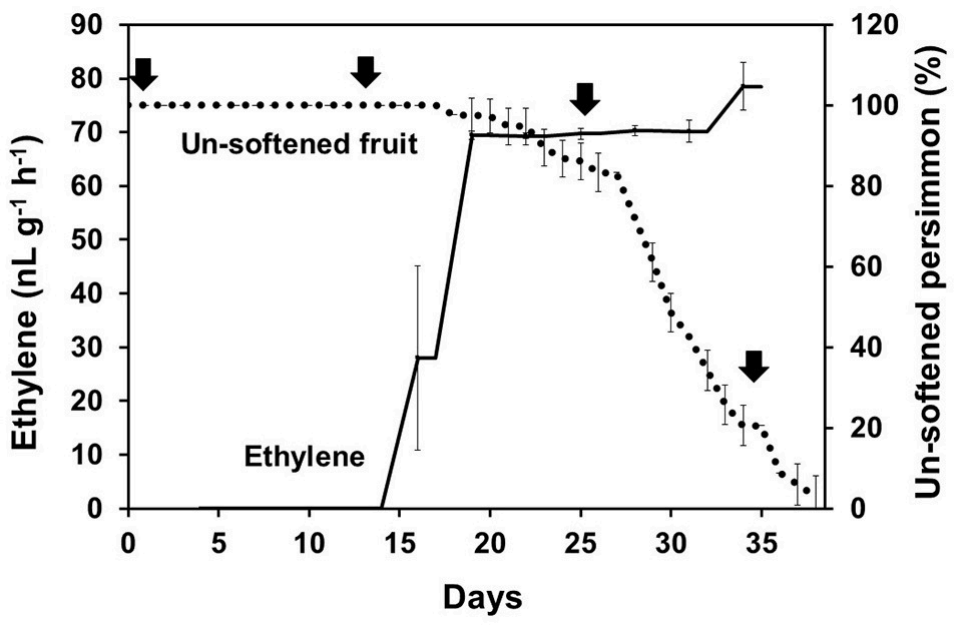
Persimmon softening and production of ethylene gas. Persimmon softening is shown in terms of unsoftened persimmon (%) with a black dotted line. Ethylene abundance is indicated with a solid black line. Arrows indicate sampling points for qRT-PCR. Error bars represent standard deviation (SD).

**Figure 5:**
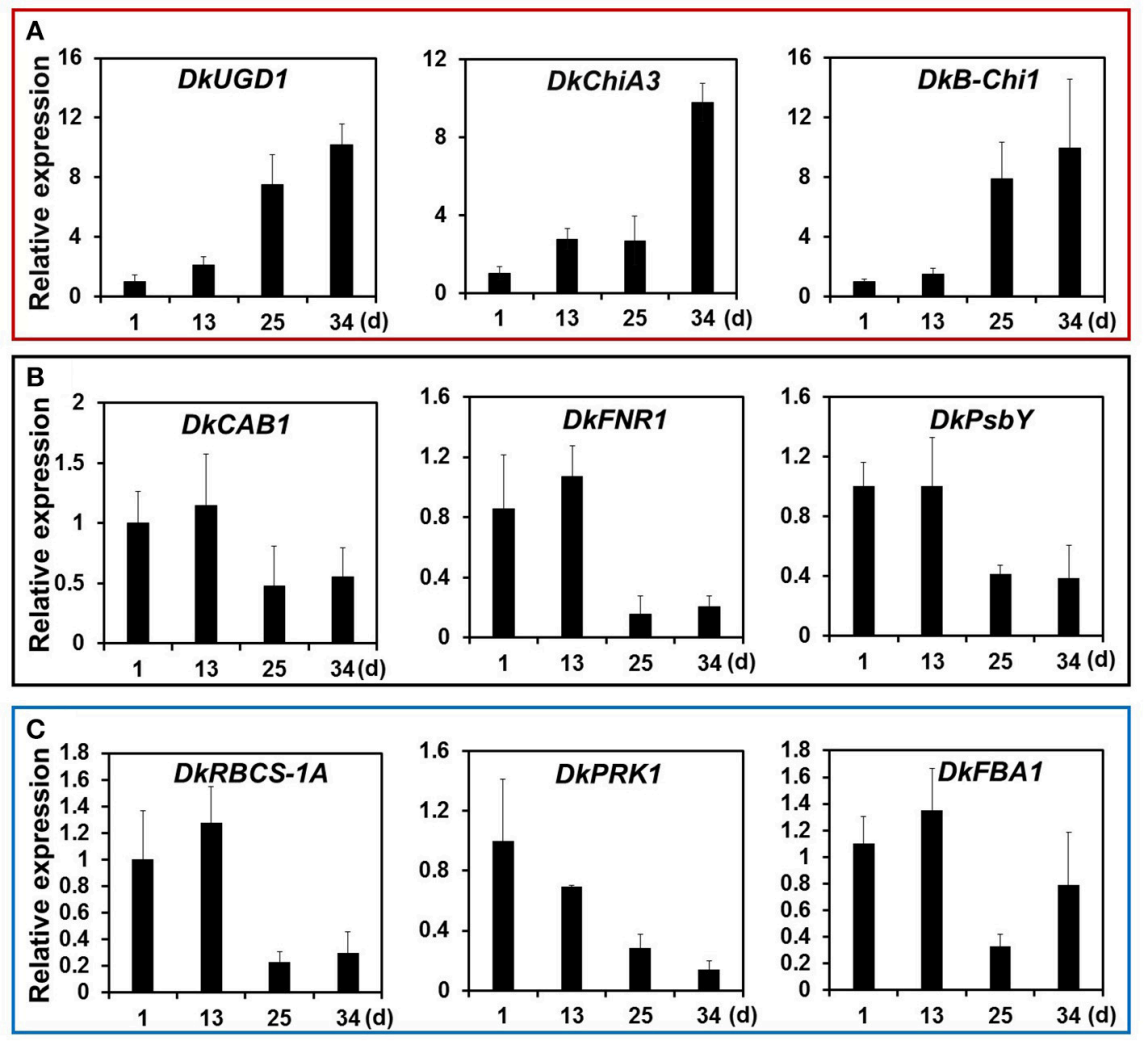
Quantitative real-time PCR analysis of representative DEGs of KEGG pathways modulated during persimmon fruit softening. **(A)** “Sugar metabolism,” **(B)** “Photosynthesis,” and **(C)** “Carbon fixation.” Transcription is shown relative to day 1 (expression level = 1) with the *DkActin* gene as an internal reference. Each qRT-PCR analysis was repeated three times. Error bars represent standard deviation (*SD*).

KEGG pathway analysis of DEGs was used to identify the major biochemical pathways modulated during persimmon softening. Carbohydrate and amino acid metabolic pathways were up-regulated in ST persimmon, as were secondary metabolite biosynthetic pathways (Table S6). Previously, starch breakdown and sucrose synthesis were observed during the rapid stage of fruit ripening and softening (Cordenunsi and Lajolo, 1995). These results support that enhancement of sugar metabolism pathways in persimmon is related to fruit softening. Differential expression of the three identified genes in the amino sugar and nucleotide sugar metabolism category during softening were verified using qRT-PCR (**Figure 5**). After harvest, samples were taken at days 1 and 13 (prior to softening onset at 16 DAH), and 25 and 34 (Figure 4). Consistent with the RNA-seq analysis, all the examined genes (*DkUGD1, DkChiA3*, and *DkB-Chi1*) were up-regulated at 25 and 34 DAH compared with 1 and 13 DAH (Figure 5A).

Photosynthetic pathways were down-regulated in ST persimmon, as were pathways involved in carbon fixation in photosynthetic organisms, carotenoid biosynthesis, and glycolysis/gluconeogenesis (Table 3; Table S6). Genes identified in the photosynthesis and carbon fixation pathways were examined using qRT-PCR. Four of the six photosynthetic genes and all five carbon fixation genes exhibited lower expression at 25 and 34 DAH than at 1 and 13 DAH (Figures 5B,C; Figures S3 and S4). Minor discrepancies between the RNA-seq and qRT-PCR results were observed for two genes, *DkPsaG* and *DkPsaD-1*(Figure S3). This discrepancy may be explained by the low expression levels and minimal differential expression observed for *DkPsaG* and *DkPsaD-1* (Table 3). Similar discrepancies between RNA-seq and qRT-PCR results were previously observed in other studies (Wang R. et al., 2017).

**Table 3:** Summary of DEGs annotated using KEGG.

**Gene (Accession)**	**FH**	**ST**	**Fold change**	**Description**	***E*-value**	**Identity**	**Species**
	**(No. of reads)^a^**						
**AMINO SUGAR AND NUCLEOTIDE SUGAR METABOLISM**							
*DkUGD1* (KX871194)	1,501	5,132	3.42	UDP-glucose 6-dehydrogenase family protein	0	94.59	*Citrus clementina*
*DkChiA3* (KX871197)	150	525	3.51	Chitinase A	1.00E-144	78.57	*Citrus clementina*
*DkB-Chi1* (KX871200)	190	2,261	11.88	Basic chitinase	1.00E-153	73.42	*Theobroma cacao*
**PHOTOSYNTHESIS**							
*DkPsbP-1* (KX871201)	2,221	254	0.11	Photosystem II subunit P-1	2.00E-120	77.82	*Arabidopsis lyrata*
*DkCAB1* (KX871202)	2,416	433	0.18	Chlorophyll A-B-binding family protein	1.00E-108	76.51	*Vitis vinifera*
*DkFNR1* (KX871203)	2,951	428	0.15	Ferredoxin-NADP(+)-oxido-reductase 1	0	89.86	*Glycine max Wm82.a2.v1*
*DkPsaD-1* (KX871204)	3,629	917	0.25	Photosystem I subunit D-1	8.00E-126	85.12	*Ricinus communis*
*DkPsaG* (KX871205)	483	52	0.11	Photosystem I subunit G	3.00E-79	78.57	*Mimulus guttatus v2.0*
*DkPsbY* (KX871206)	3,128	752	0.24	Photosystem II BY	2.00E-43	79.05	*Populus trichocarpa*
**CARBON FIXATION IN PHOTOSYNTHETIC ORGANISMS**							
*DkRBCS-1A* (KX871207)	2,835	129	0.05	Ribulose bisphosphate carboxylase small chain 1A	3.00E-53	77.14	*Ricinus communis*
*DkPRK1* (KX871208)	1,848	254	0.14	Phosphoribulokinase	0	91.87	*Populus trichocarpa*
*DkFBA1* (KX871209)	1,201	86	0.07	Fructose-bisphosphate aldolase 2	0	93.23	*Solanum lycopersicum*
*DkGAPA* (KX871210)	1,219	157	0.13	Glyceraldehyde 3-phosphate dehydro-genase A subunit 2	0.00E+00	90.17	*Theobroma cacao*
*DkGAPB* (KX871211)	791	68	0.09	Glyceraldehyde-3-phosphate dehydrogenase B subunit	0.00E+00	90.87	*Malus domestica*

a *Average of three replicates*.

### Identification of ethylene-related DEGs in persimmon

**Figure 6:**
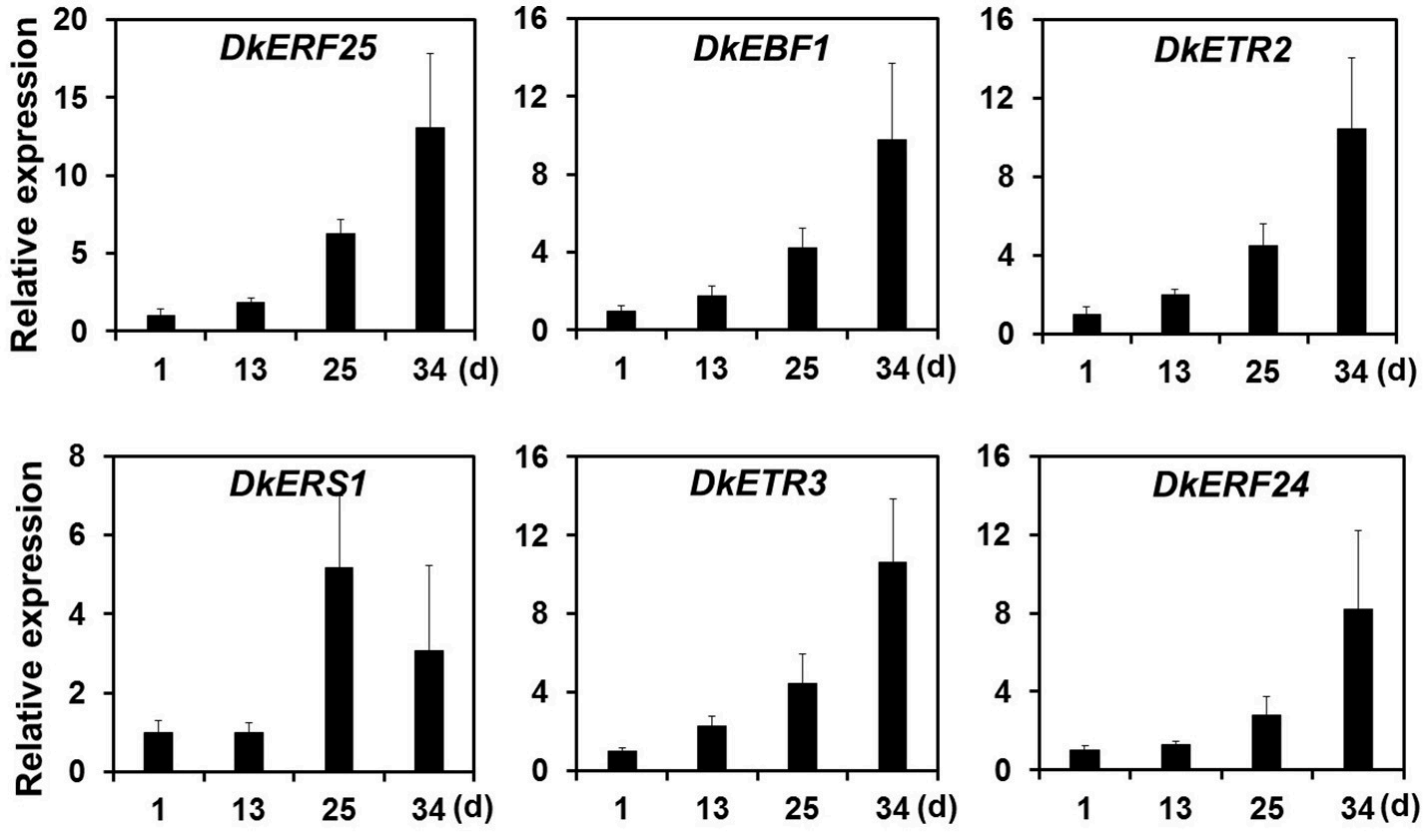
Quantitative real-time PCR analysis of DEGs involved in ethylene-perception and signaling during persimmon fruit softening. Transcription is shown relative to day 1 (expression level = 1) with the *DkActin* gene as an internal reference. Each qRT-PCR analysis was repeated three times. Error bars represent standard deviation (*SD*).

**Figure 7:**
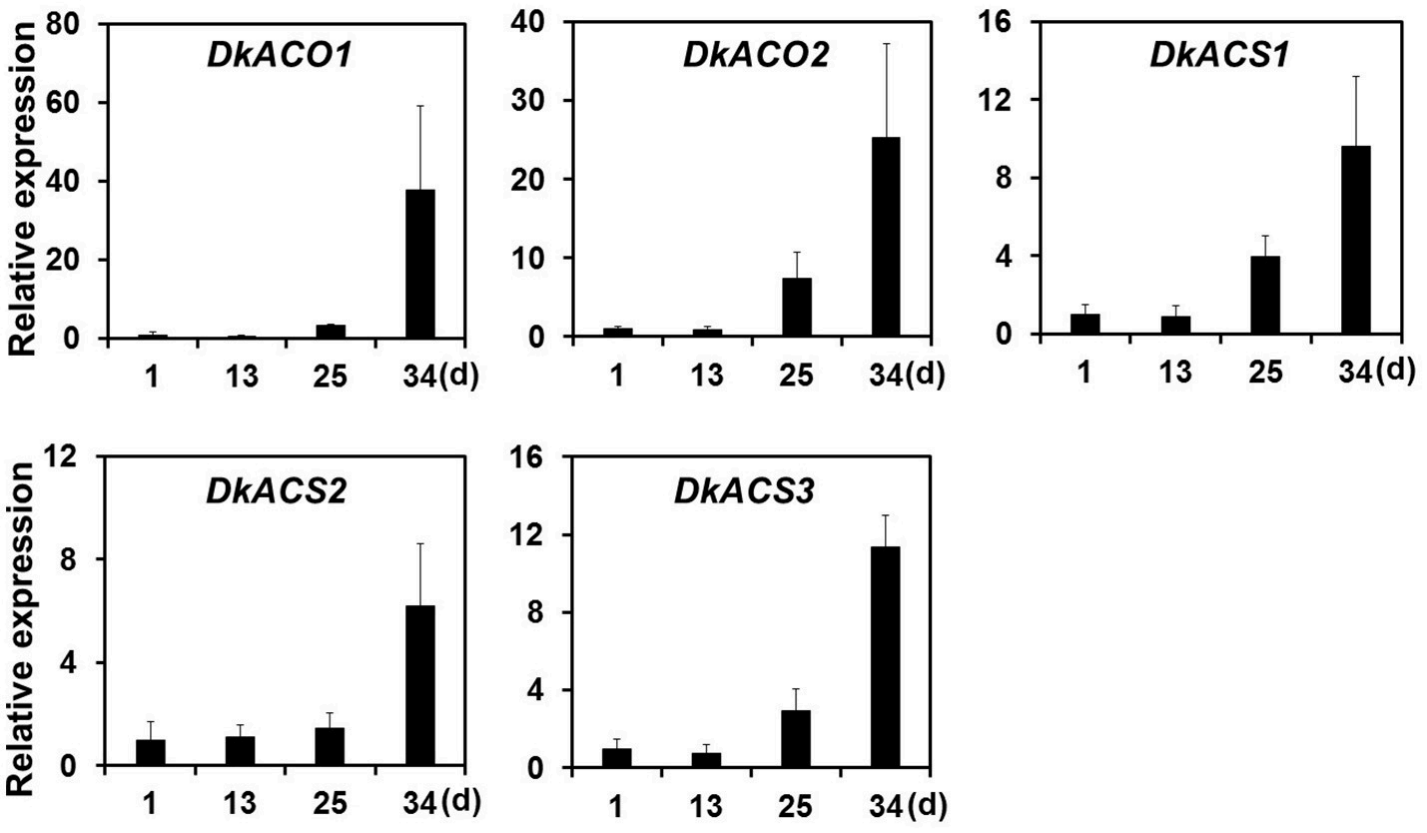
Quantitative real-time PCR analysis of ethylene-biosynthesizing genes during persimmon fruit softening. Transcription is shown relative to day 1 (expression level = 1) with the *DkActin* gene as an internal reference. Each qRT-PCR analysis was repeated three times. Error bars represent standard deviation (*SD*).

Ethylene, which is produced by persimmon fruits during maturation, induces expression of softening-related genes (Tucker and Brady, 1987; Sitrit and Bennett, 1998). As ethylene is a major effector of fruit softening, several previous studies have examined the effects of reducing ethylene impacts on persimmon by treatment with ethylene inhibiting chemicals such as 1-MCP (Nakano et al., 2003; Vilas-Boas and Kader, 2007). However, only a few genes involved in ethylene production or in the response to ethylene during softening in persimmon were discovered (Nakano et al., 2003; Pang et al., 2007; Min et al., 2014). In this study, ethylene gas production during softening was quantitated using gas chromatography. Ethylene gas was first detected 16 DAH and corresponded to the onset of fruit softening (Figure 4). DEG analysis revealed six transcripts that were up-regulated in ST compared with FH and encoded predicted ethylene-related genes (Table 4; Table S5). These genes included ethylene response factor, ethylene receptor, and EIN3-binding F-BOX protein. Two of the transcripts, namely, *DkETR2* and *DkERS1*, were identified as *D. kaki* transcripts during BLASTx annotation, indicating their previous identification in *D. kaki* (Pang et al., 2007). The remaining four transcripts (*DkERF25, DkEBF1, DkERF24*, and *DkETR3*) were newly identified in *D. kaki* (Table 4). Up-regulation of the ethylene-related DEGs during softening was verified at 1, 13, 25, and 34 DAH using qRT-PCR analysis (Figure 6). Consistent with RNA-seq data, expression of all six genes was elevated at 25 and 34 DAH compared with 1 and 13 DAH (Figure 6). Interestingly, the transcripts involved in ethylene biosynthesis were not detected in the DEG analysis (Table S5). Only *DkACO1* (gene ID: 1SL019260t0001), but not the others, was identified in the RNA-seq assembly database (Table S2). Nevertheless, since the ethylene biosynthesis genes, ACC synthase (*DkACS1*, -*2*, and -*3*) and ACC oxidase (*DkACO1* and -*2*), have been previously reported (Nakano et al., 2003; Ortiz et al., 2006), we determined the expression patterns of the genes by qRT-PCR analysis (Figure 7). All five genes showed significant increase at days 25 and 34 after harvest, while no distinct modulation of expression until 13 days (Figure 7), consistent with ethylene production pattern (Figure 4).

**Table 4:** Transcripts encoding predicted ethylene-related genes.

**Gene (Accession)**	**FH**	**ST**	**Fold change**	**Description**	***E*-value**	**Identity**	**Species**
	**(No. of reads)^a^**						
*DkERF25* (KX871212)	37	293	7.85	ethylene response factor 1-like protein ERF1–2	2.00E-84	57.98	*Dimocarpus longan*
*DkEBF1* (KX871213)	463	3,106	6.71	EIN3-binding F-box protein 1-like	0	70.72	*Sesamum indicum*
^*^DkETR2 (AB243790)	503	3,691	7.34	Ethylene receptor	0	99.34	*Diospyros kaki*
^*^DkERS1 (AB164038)	572	4,070	7.11	Ethylene receptor	0	99.84	*Diospyros kaki*
*DkERF24* (KX871216)	119	529	4.46	Ethylene response factor 14	1.00E-52	65.52	*Actinidia deliciosa*
*DkETR3* (KX871217)	1576	7,826	4.97	Ethylene receptor 2	0	73.9	*Paeonia lactiflora*

a *Average of three replicates*.

* *Previously identified genes*.

## Discussion

The ability to delay fruit ripening is important in the food industry to prevent fruit decay and prolong storage time. Persimmon production is limited to a small number of geographical locations, and persimmon softening hampers successful fruit export (FAOSTAT, 2013). Persimmon is hexaploid (2*n* = 6*x* = 90) and does not have a sequenced genome, both of which hamper the study of genes related to fruit softening. Although NCBI contains data from previous studies for 14,482 ESTs, 1,238 nucleotides, and 755 protein sequences of *D. kaki* (as of January 2017), little is known regarding the biochemical and physiological pathways of relevance to persimmon softening. *De novo* assembly is an established technique for developing genome-level resources for non-model organisms, including plant species (Vera et al., 2008; Meyer et al., 2009; Der et al., 2011). In this study, *de novo* assembly of RNA-seq data was used to identify and compare transcripts from FH and ST persimmon peel samples. Genes related to fruit softening were identified using differential expression analysis.

Ripening is a major determinant of fruit quality. Traits such as fruit color, texture, flavor, and aroma are affected by ripening and influence final fruit quality (Miszczak et al., 1995; Brummell et al., 2004; Cherian et al., 2014). During ripening, changes in gene expression or protein regulation lead to physiological and biochemical effects such as ethylene biosynthesis, pigmentation synthesis, chlorophyll degradation, cell wall degradation, and organic acid accumulation (Brady, 1987; Cherian et al., 2014).

KEGG pathway analysis showed that the up-regulated pathways during persimmon softening include “amino sugar and nucleotide sugar metabolism.” In this pathway, transcription of basic chitinase, chitinase A, and UDP-glucose 6-dehydrogenase was up-regulated during softening. Expression of these genes was also found in previous studies. For example, EST analysis identified basic chitinase and endochitinase gene expression at a late stage of fruit development in persimmon (Nakagawa et al., 2008). Expression of the *MaECHI1* gene encoding banana endochitinase also increased markedly during banana ripening and after exogenous ethylene treatment (Liu et al., 2012). Functionally, chitinase hydrolyzes colloidal chitin into chito-oligomers, including chitotriose and chitobiose, and its monomer N-acetylglucosamine (Guthrie et al., 2005). The major function of endochitinase is thought to be hydrolysis of chitin and formation of a structural polysaccharide, resulting in modification of cell wall morphogenesis (Sahai and Manocha, 1993; Arakane and Muthukrishnan, 2009). Chitinase and endochitinase activities would therefore have the physical effect of loosening cell walls. UDP-glucose 6-dehydrogenase was identified as a member of the sucrose metabolism pathway (Table 3). UDP-glucose 6-dehydrogenase is a pectin-degrading enzyme (Wei et al., 2015). Fruit firmness is closely related to soluble pectin content, and up-regulation of UDP-glucose 6-dehydrogenase expression may thus lead to fruit softening (Fischer and Bennett, 1991). Expression of UDP-glucose 6-dehydrogenase was found to be increased in melon fruits at a late stage of maturation (Guo et al., 2017). Additional KEGG pathways, including those related to sucrose metabolism (“starch and sucrose metabolism” and “galactose metabolism”) and amino acid metabolism (“alanine, aspartate, and glutamate metabolism,” “glycine, serine, and threonine metabolism,” and “tryptophan metabolism”), were shown to be up-regulated in the fruit peels of ST persimmon (Table S6). The up-regulation of sugar metabolism was reported to be consistent with the increased sugar content seen in persimmon and other fruits, such as apple and tomato, during ripening (Lee et al., 2007; Gautier et al., 2008). Biosynthetic pathways for secondary metabolites such as terpenoids, steroids, carotenoids, and alkaloids were also significantly up-regulated in fruit peels of ST persimmon (Table S6), indicating that fruit softening is a complex process accompanied by the dynamic degradation and generation of different metabolites.

KEGG analysis also showed that genes related to “photosynthesis” and “carbon fixation in photosynthetic organisms” decreased in expression as persimmons matured. Decreased photosynthesis during ripening was reported for other fruits such as apple, tomato, and strawberry (Paul and Driscoll, 1997; Lee et al., 2007; Wang Q. H. et al., 2017). During the transition from green to red fruits (chloroplast/chromoplast differentiation), mRNA levels for photosynthesis-specific polypeptides decreased in tomato (Piechulla et al., 1985). Tomato fruits undergo a physiological transition during the differentiation of photosynthetically active chloroplasts into non-photosynthetic chromoplasts, and it was suggested that this transition was coupled to changes in gene expression (Lytovchenko et al., 2011). RNA-seq analysis of strawberry also showed decreased expression of genes in the “photosynthesis” and “carbon fixation” pathways during ripening. Expression of the genes encoding the components of photosystems I and II decreased during persimmon softening (Figure 5B; Table 3). Similarly, chlorophyll content of photosystems I and II decreased significantly during rice leaf senescence (Tang et al., 2005). Reduced photosynthetic activity may lead to reduction of carbon fixation, but expression of genes involved in carbon fixation has not been well-characterized with respect to fruit ripening. This study revealed the identities of genes involved in carbon fixation, which was down-regulated during persimmon fruit softening (Figure 5C and Figure S4; Table 3).

After harvest, persimmon produces ethylene gas, which may stimulate fruit softening in climacteric fruits. Persimmons undergo stress following harvest due to loss of water and nutrients provided by the parent tree (Nakano et al., 2003). In persimmon, water loss induces expression of several ethylene biosynthesis genes, including ACC synthase (*DkACS1*, -*2*, and -*3*) and ACC oxidase (*DkACO1* and -*2*). Expression of these genes is temporally and spatially regulated during fruit softening (Nakano et al., 2003). Consistently, our study also detected up-regulation of all the five ethylene biosynthesis genes by qRT-PCR analysis (Figure 7). However, these genes were undetected in assembled database except *DkACO1* (persimmon1SL019260t0001), possibly, due to low expression levels at different sampling times (Table S2). *DkACO1* was then cut off in our DEG analysis because of poor consistency among replicates. In contrast, ethylene response factor, ethylene receptor, and EIN3-binding F-BOX protein were consistently detected. Previous study had shown that ethylene biosynthesis were induced mainly in the calyx of persimmon and subsequently diffused to other fruit tissues (Nakano et al., 2003). Our results support that the peel of persimmon fruit is a site of ethylene perception as well as a site of ethylene biosynthesis.

Twelve ethylene response factors (*DkERF11-22*) were previously identified from persimmon (Min et al., 2014). In this study, *DkERF21* and *DkERF22* (gene IDs: 1SL019317t0001 and 1SL020456t0001) were up-regulated during softening (Table S2). The ethylene receptor genes *DkERS1, DkETR1*, and *DkETR2* were identified in persimmon as homologs of *Arabidopsis* genes (Pang et al., 2007). Expression of *DkERS1* and *DkETR2* correlated with ethylene quantities during fruit softening, and expression was enhanced after ethylene treatment (Pang et al., 2007). The present study also found that *DkERS1* and *DkETR2* were up-regulated during persimmon fruit softening (Figure 6). In addition, four new ethylene-related transcripts (*ERF25, EBF1, ETR3*, and *ERF24*) that were up-regulated during persimmon softening were identified in this study. These genes may be good targets for future studies of persimmon softening.

This study investigated the transcriptional events occurring during persimmon fruit softening by identifying genes implicated in increased sugar metabolism, decreased photosynthetic capability, and increased ethylene production and ethylene-related functions. This transcriptome analysis provides baseline information on the identity and modulation of genes involved in softening of persimmon fruits.

## Author contributions

SR conceived, designed, supervised the research, and made final revision of the manuscript. JJ collected samples, generated data, and wrote a draft of the manuscript. SC assisted data analysis. SJ confirmed some data by repeating the qRT-PCR assay. BC revised the manucript. GA were involved in the sample collection. All authors read and approved the final manuscript.

### Conflict of interest statement

The authors declare that the research was conducted in the absence of any commercial or financial relationships that could be construed as a potential conflict of interest.
