# Supplementary material for: A Transcriptome Approach Toward Understanding Fruit Softening in Persimmon
Source: Front Plant Sci. 2017 Sep 12;8:1556. doi: 10.3389/fpls.2017.01556 (PMC5601038; doi:10.3389/fpls.2017.01556)
Supplement: Supplementary file 1 [file Presentation1.PDF]

Supplementary Material

A transcriptome approach towards understanding fruit softening in persimmon

Jihye Jung, Sang Chul Choi, Sunghee Jung, Byung-Kwan Cho, Gwang-Hwan Ahn, Stephen Beungtae Ryu*

*** Correspondence:** S.B. Ryu: sbryu@kribb.re.kr

Supplementary Figures and Tables

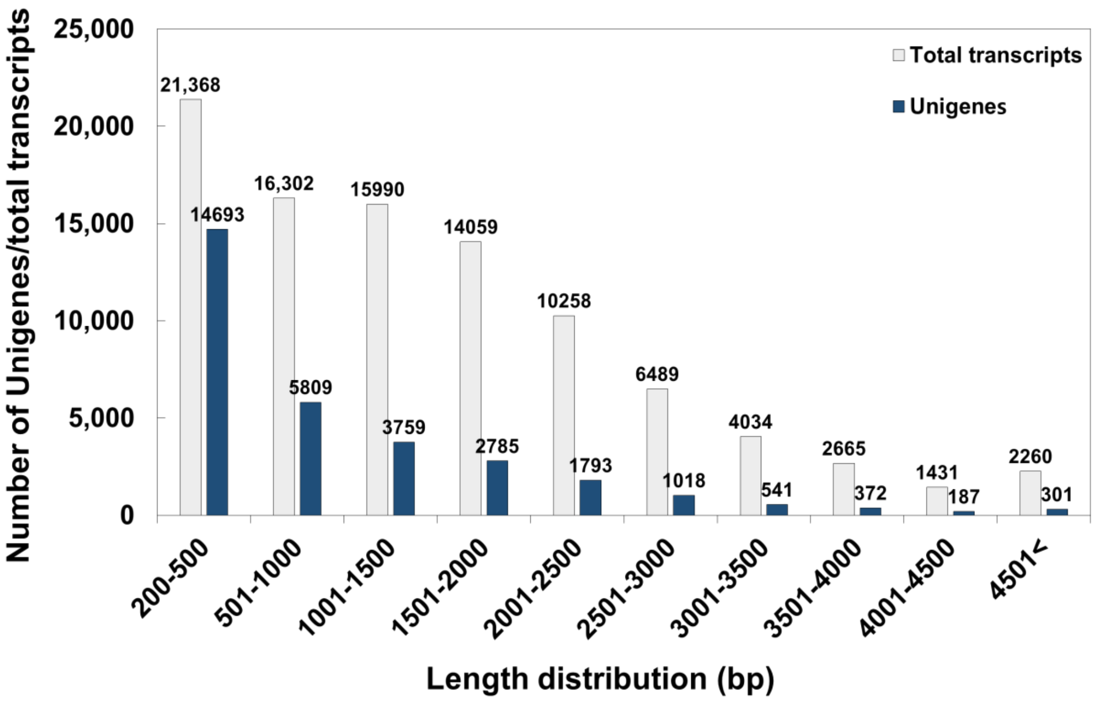

Figure S1. Length distribution of assembled total transcripts and unigenes. The numbers of total transcripts and unigenes whose lengths fell within the specified bins are shown**.**

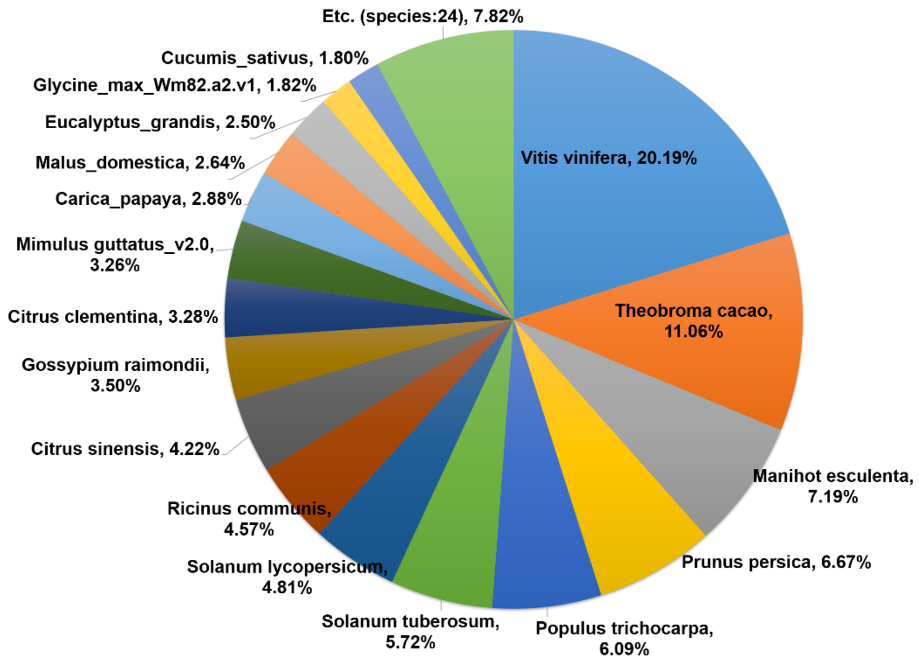

Figure S2. Annotated species and percentage of unigenes in the Phytozome database.

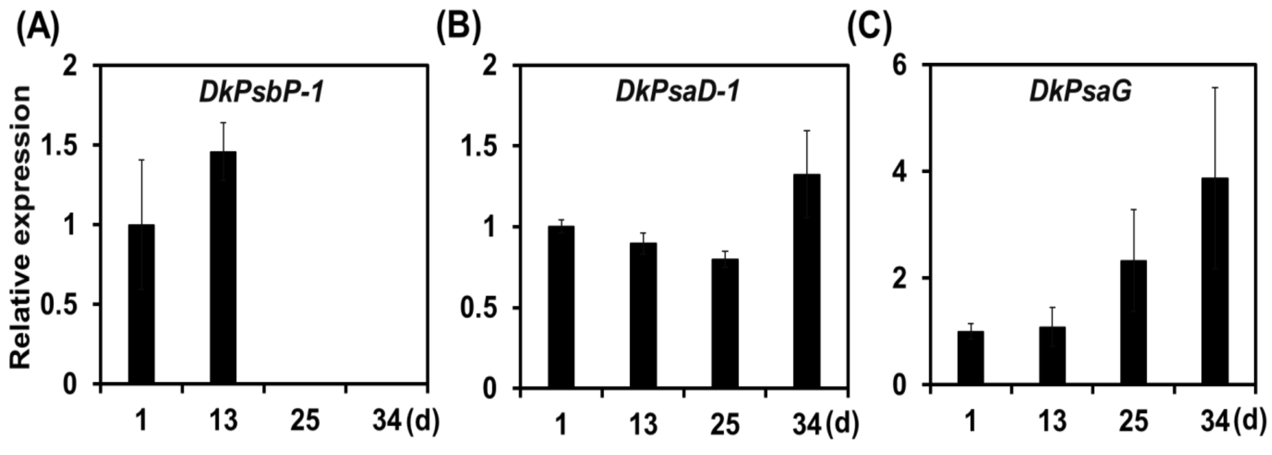

Figure S3. qRT-PCR analysis of genes annotated in the 'photosynthesis' pathway. (A-C) Gene expressions were examined at days 1, 13, 25, and 34 after harvest. Transcription is shown relative to day 1 (expression level = 1) with the *DkActin* gene as an internal reference. Each qRT-PCR analysis was repeated three times. Error bars represent standard deviation (SD).

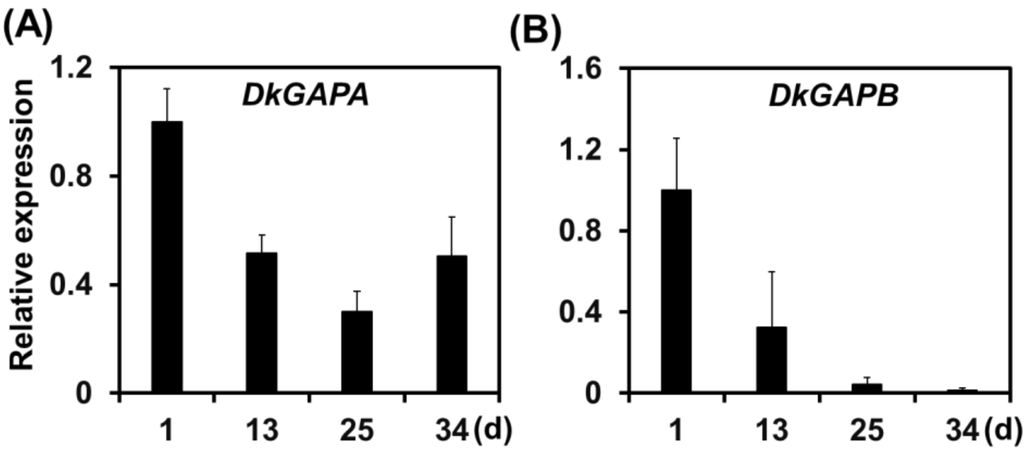

Figure S4. qRT-PCR analysis of genes annotated in the 'carbon fixation' pathway. (A and B) Gene expressions were examined at days 1, 13, 25, and 34 after harvest. Transcription is shown relative to day 1 (expression level = 1) with the *DkActin* gene as an internal reference. Each qRT-PCR analysis was repeated three times. Error bars represent standard deviation (SD).

Table S1. Raw sequencing data of freshly harvested and softened samples

Sample name	Sample description	Number of reads	Average length (bp)	Total length (bp)
FH1_R1	Freshly harvested (FH)	25,825,436	101	2,608,369,036
FH1_R2		25,825,436	101	2,608,369,036
FH2_R1		23,543,549	100	2,362,326,259
FH2_R2		23,543,549	100	2,362,660,295
FH3_R1		22,826,094	100	2,290,820,012
FH3_R2		22,826,094	100	2,291,045,500
ST1_R1	Softened (ST)	22,943,990	101	2,317,342,990
ST1_R2		22,943,990	101	2,317,342,990
ST2_R1		26,010,033	100	2,610,070,508
ST2_R2		26,010,033	100	2,610,242,351
ST3_R1		23,431,735	100	2,351,482,034
ST3_R2		23,431,735	100	2,351,865,292
Total		**289,161,674**	101	**29,081,936,303**

Tables S2–S6 are presented in separate files.

Table S7. Primers used for qRT-PCR analysis

Unigene	Sequence (5'-3')
DkActin (AB473616)	F: CATGGAGAAAATCTGGCATCATAC<br>R: GAAGCACTGGGTGCTCTTCTG
DkUGD1	F: ACCACCGTGAAGCAAGTTTC<br>R: ATCTCCCTCAGCTTATCCGC
DkChiA3	F: TCTTCTGGCACCTGTACTGG<br>R: TCCATCTAGAACGGCGTCTC
DkB-Chi1	F: TGCGTGGGGATATTGCTTTG<br>R: GCTTTGCCCGCTAAACCATA
DkPsbP-1	F: CCCACAAACAGGACGAACAA<br>R: CACTTCCTTGCTTGGGTTCC
DkCAB1	F: CCCATTTACGAAGCTGAGCC<br>R: AGCTCCTTTTCCGGTGATGA
DkFNR1	F: CAATTCCGGTTCCAGTTGCA<br>R: AGGCTCGTGTACACCAATGA
DkPsaD-1	F: TACCCCAACTCTCTCCACCT<br>R: GGCCTCCTTCGTCTCCTTTA
DkPsaG	F: AAACCCTCCTCCGTCAAACT<br>R: TAACGTACTCCTTGGCCCTG
DkPsbY	F: AGGGTCGGATTGTCATGGTT<br>R: AATCCGGTCCGATCAGATCC
DkRBCS-1A	F: TACTCGTCATGGCTTCCTCC

	R: CACACCTTCATGCACTGGAC
DkPRK1	F: CTCAAGACCTTCCCCTCGTT<br>R: TTCAGAGGGACATGGCAGAG
DkFBA1	F: ATTCCCTTCTTGGCCTCCTC<br>R: TCTGGTGGGCAATCAGAAGT
DkGAPA	F: CGTACTTGAGGAGGTGGGAG<br>R: TCGTTGCATTCCAGACCTCT
DkGAPB	F: AGGTGCCAAGAGTTGAGTCA<br>R: CTGCCCAACTTACAGCCAAG
DkERF25	F: CGCCGGATAAACAACCCTTT<br>R: TGCCACTGAAGGAGAACGAT
DkEBF1	F: TCAAGACGGAGGAGGTTGAC<br>R: TCCCCGAATCTGACTGCTTT
DkETR2<br>(AB243790)	F: AGTTCCTCATTGCTCCACCA<br>R: CTTGCTGCTTCTAGTGCTCG
DkERS1<br>(AB164038)	F: CTGTTGGTGATGAGAAGCGG<br>R: ACCACAGCCAGAATCCTTCA
DkERF24	F: GCCCTTGCCTATGATGAAGC<br>R: ATGGCATGATGAAGCTGCTG
DkETR3	F: TCGGTCTCCTCATTCCATCG<br>R: GGCTATTTCTCACGCCACAG
[1]*DkACS1*<br>(AB073005)	F: CCACTGCGACGAACCGGGTTGG<br>R: TCAGGCTCGCGGCCATCGAACG

[1]*DkACS2* (AB073006)	F: AGAATCCGGACGTTCGTGGATGA<br>R: AAGCATAGGGGAGTGAGGCGACAAC
[1]*DkACS3* (AB073007)	F: CTCAACGTCTCGCCGGGAGTGTCTT<br>R: GAGGGGACATCATGGCAATGTCATC
[1]*DkACO1* (AB073008)	F: TGGCAATGATGCTGTTATCTATC<br>R: CGAACTATTACAAATAACATGTGTC
[1]*DkACO2* (AB073009)	F: CAGCGACGCAGTGATTTATCCAG<br>R: CAGAGGGCTTGGCTTAGACTGTGGC

F, forward; R, reverse.

[1]The primers of ethylene biosynthesis genes were obtained from Ortiz et al. (2006).

Table S8. NCBI accession numbers of transcripts

Gene name	NCBI accession number	Gene ID
DkUGD1	KX871194	persimmon1SL009272t0004
DkChiA3	KX871197	persimmon1SL020061t0002
DkB-Chi1	KX871200	persimmon1SL018182t0002
DkPsbP-1	KX871201	persimmon1SL003093t0001
DkCAB1	KX871202	persimmon1SL021690t0003
DkFNR1	KX871203	persimmon1SL006336t0002
DkPsaD-1	KX871204	persimmon1SL022901t0001
DkPsaG	KX871205	persimmon1SL023056t0001
DkPsbY	KX871206	persimmon1SL017030t0002
DkRBCS-1A	KX871207	persimmon1SL019810t0001
DkPRK1	KX871208	persimmon1SL004815t0001
DkFBA1	KX871209	persimmon1SL005882t0001
DkGAPA	KX871210	persimmon1SL004468t0002
DkGAPB	KX871211	persimmon1SL004784t0001
DkERF25	KX871212	persimmon1SL005151t0005
DkEBF1	KX871213	persimmon1SL007183t0009
DkERF24	KX871216	persimmon1SL007733t0004
DkETR3	KX871217	persimmon1SL001994t0004
